# Interaction of macrophages with apoptotic cells inhibits transdifferentiation and invasion of lung fibroblasts

**DOI:** 10.18632/oncotarget.22737

**Published:** 2017-11-28

**Authors:** Yong-Bae Kim, Young-So Yoon, Youn-Hee Choi, Eun-Mi Park, Jihee Lee Kang

**Affiliations:** ^1^ Tissue Injury Defense Research Center, College of Medicine, Ewha Womans University, Seoul 07985, Korea; ^2^ Department of Physiology, College of Medicine, Ewha Womans University, Seoul 07985, Korea; ^3^ Department of Pharmacology, College of Medicine, Ewha Womans University, Seoul 07985, Korea

**Keywords:** apoptotic cells, macrophages, lung fibroblasts, myofibroblast, invasion

## Abstract

The invasion of activated fibroblasts is a key mechanism of tissue fibrosis pathology. The recognition and uptake of apoptotic cells can induce the anti-fibrogenic programming of macrophages. We demonstrate that after interacting with apoptotic cells, macrophages secrete bioactive molecules that antagonize TGF-β1-induced increases in myofibroblast (fibroproliferative) phenotypic markers and reduce the enhanced invasive capacity of TGF-β1- or EGF-treated mouse lung fibroblasts (MLg). Furthermore, numerous treatment strategies prevented the anti-fibrotic effects of conditioned media, including transfection of macrophages with COX-2 or RhoA siRNAs or treatment of MLg cells with receptor antagonists for prostaglandin E_2_ (PGE_2_), PGD_2_, or hepatocyte growth factor (HGF). Additionally, administration of apoptotic cells *in vivo* inhibited the bleomycin-mediated invasive capacity of primary fibroblasts, as well as adhesion and extracellular matrix protein mRNA expression. These data suggest that the anti-fibrogenic programming of macrophages by apoptotic cells can be used as a novel tool to control the progressive fibrotic reaction.

## INTRODUCTION

Pulmonary fibrosis is a progressive and lethal disease characterized by the excessive deposition of extracellular matrix (ECM) components, such as collagens I and III, fibronectin, and lamin, in the lung parenchyma and distal airspace. In idiopathic pulmonary fibrosis (IPF), which is the most common and has the worst prognosis of all idiopathic interstitial diseases, the excessive production and deposition of the ECM occurs in areas where apoptosis-resistant, activated fibroblasts have accumulated [1]. Although the pathogenic mechanisms leading to IPF have not been fully elucidated, dysregulation of apoptosis and ageing appears to be the major driving forces [2, 3]. Both the incidence and prevalence of IPF markedly increase with advancing age [3, 4]. In particular, apoptosis of alveolar epithelial type II cells in Hermansky-Pudlak syndrome–associated interstitial pneumonia in both mice and humans may represent a prominent reason for development of lung fibrosis [5]. This excessive fibroblast accumulation depends on fibroblast migration to the site of tissue injury and invasion of the ECM [6]. Furthermore, fibroblast invasion from the lung interstitium into the airspaces is a general feature of fibrosis progression [4, 7].

Apoptotic cell clearance by tissue macrophages and non-professional phagocytes is an essential process in maintaining tissue health and function. The interaction of apoptotic cells with phagocytic cells induces the release of tissue maintenance factors, such as vascular endothelial growth factor (VEGF), hepatocyte growth factor (HGF), and prostaglandin E2 (PGE_2_), which are critical for tissue repair and suppression of the damaging fibroproliferative response [8–11]. In a murine model of pulmonary fibrosis, we previously demonstrated that a single exposure to apoptotic cells mediates an anti-inflammatory and anti-fibrotic effect *via* persistent up-regulation of pro-resolving cytokines, such as HGF, PGE_2_, and PGD_2_ [12–15]. Importantly, many studies provide evidence that these paracrine signals inhibit the fibrotic response via inhibition of the fibroblast to myofibroblast transition [16]. However, it is unclear if the prostaglandin and HGF pathways prevent fibroblast activation through the enhanced apoptotic cell recognition and clearance of macrophages.

In the present study, we evaluated the influence of apoptotic cells in driving an anti-fibrogenic macrophage program for controlling fibroblast activation. Using an *in vitro* co-culture system, we determined that macrophages exposed to apoptotic cells secrete paracrine factors (PGE_2_, PGD_2_, and HGF) that modulate lung fibroblast activation and invasion. In particular, we demonstrated an anti-invasive effect of *in vivo* apoptotic cell administration on primary lung fibroblasts after bleomycin treatment.

## RESULTS

### Interaction of macrophages with apoptotic cells inhibits myofibroblast phenotypic markers

**Figure 1:**
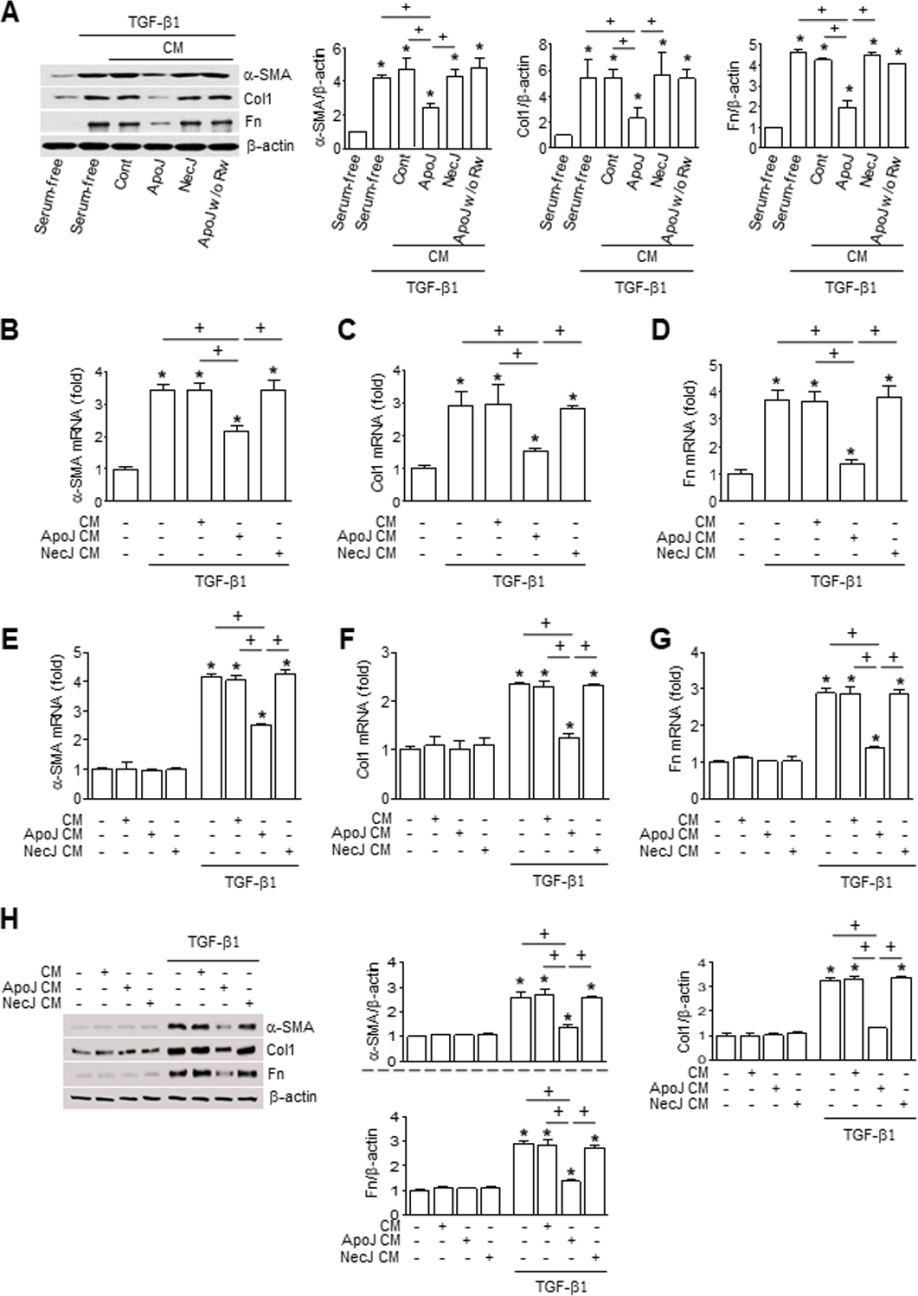
Conditioned medium from macrophages exposed to apoptotic cells reduces myofibroblast phenotypic marker in lung fibroblasts RAW 264.7 cells were stimulated with apoptotic (ApoJ) or necrotic (NecJ) Jurkat cells for 20 h. Conditioned medium (CM) was added to MLg cells (**A**–**D**) or primary mouse lung fibroblasts (**E**–**G**) in the absence or presence of 10 ng/ml TGF-β1 for 24 h. (**H**) CM from primary mouse BMDM was added to MLg cells in the absence or presence of 10 ng/ml TGF-β1 for 24 h. A and H Immunoblots of total cell lysates were performed with anti-α-SMA, type 1 collagen α2 (Col1), or fibronectin (Fn) antibodies. Right: Densitometric analysis of the indicated myofibroblast phenotypic markers’ relative abundances. (B–G) The amount of myofibroblast phenotypic markers’ mRNA in MLg cell or primary lung fibroblasts samples was analyzed by real-time PCR and normalized to that of *Hprt* mRNA. Values represent the mean ± s.e.m. of three independent experiments. ^*^P < 0.05; compared with control; ^+^*P* < 0.05 as indicated.

Transforming growth factor-β (TGF-β) is regarded as the key cytokine driving the up-regulation of collagen synthesis, epithelial–mesenchymal transition (EMT), and myofibroblast transdifferentiation via Smad or non-Smad signaling pathways [17]. Therefore, we examined whether the interaction of macrophages and apoptotic cells can counteract the TGF-β-induced fibroblast activation leading to ECM deposition in organ fibrosis. Murine macrophage cells (RAW 264.7) were exposed to apoptotic Jurkat T lymphocyte cells (ApoJ) for 20 hours and this conditioned medium (CM) was added to mouse lung fibroblasts (MLg cells) in the absence or presence of TGF-β1. Treatment with the ApoJ-exposed CM for 24 hours reduced the TGF-β1-induced increases in protein and mRNA expression of myofibroblast (fibroproliferative) phenotypic markers, including α-SMA, type 1 collagen α2, and fibronectin (Figure 1A–1D). However, the inhibitory effect of the ApoJ-exposed CM was not observed with CM derived from RAW 264.7 co-culture with control or necrotic Jurkat cells (NecJ-exposed CM). The CM from RAW 264.7 cells exposed to other apoptotic cell types, such as human HeLa epithelial cells and mouse thymocytes, also inhibited TGF-β1-induced activation of MLg cells (Supplementary Figure 1A–1B). We next confirmed the inhibitory effect of the ApoJ-exposed CM on TGF-β1-induced activation of primary mouse lung fibroblasts (Figure 1E–1G). In addition, we examined interaction between primary isolated murine bone marrow-derived macrophages (BMDM) cultured in the presence of granulocyte macrophage colony-stimulating factor (GM-CSF) and apoptotic or necrotic cells for 20 h. Similar to the CM from ApoJ-exposed RAW 264.7, the CM derived from ApoJ-exposed BMDM substantially inhibited TGF-β1-induced fibroblast activation (Figure 1H). This inhibitory effect was also not observed with CM derived from BMDM co-culture with control or necrotic Jurkat cells.

**Figure 2:**
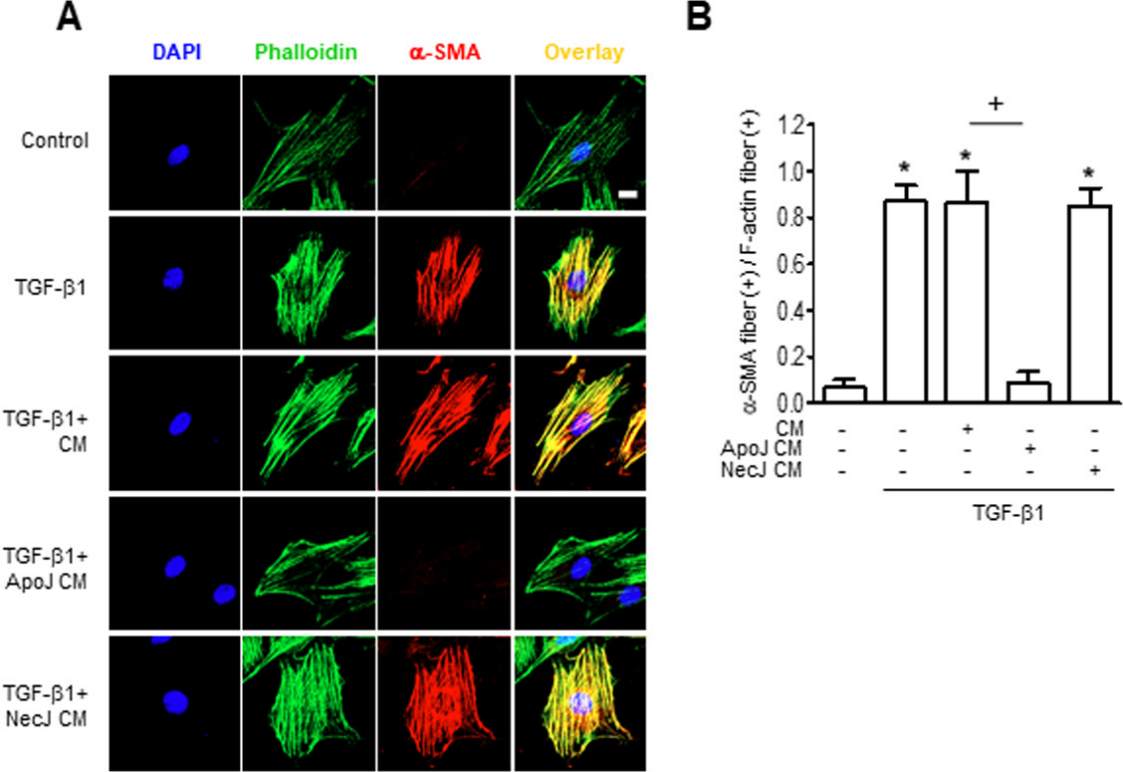
Conditioned medium from RAW 264.7 cells exposed to apoptotic cells suppresses TGF-β1 promotion of α-SMA stress fibers RAW 264.7 cells were stimulated with apoptotic (ApoJ) or necrotic (NecJ) Jurkat cells for 20 h. Conditioned medium (CM) was added to MLg cells in the absence or presence of 10 ng/ml TGF-β1 for 24 h. (**A**) Cells were co-stained for F-actin (phalloidin, green) and α-SMA (red). Scale bar: 20 μm. Results are representative of three independent experiments. (**B**) The mean ratio (± s.e.m. of three independent experiments) of α-SMA stress fiber (+) fibroblasts over all F-actin (+) fibroblasts is presented in the bar graph. ^*^P < 0.05; compared with control; ^+^*P* < 0.05 as indicated.

Myofibroblasts gain enhanced contractile activity upon incorporation of α-SMA into their actin cytoskeleton [18]. Therefore, we validated α-SMA expression in our model by assessing α-SMA recruitment to actin stress fibers. Consistent with the Western blot data, untreated MLg cells showed only weak cytosolic α-SMA expression by immunofluorescence staining. However, α-SMA staining (red) increased substantially within 24 h of TGF-β1 treatment and was predominantly co-localized with phalloidin-labeled stress fibers (green) (Figure 2A). Moreover, the percentage of fibroblasts with α-SMA-containing stress fibers increased with the addition of TGF-β1 treatment (Figure 2B). The CM from ApoJ-exposed RAW 264.7 cells inhibited TGF-β1-induced increase in α-SMA-containing stress fibers, whereas the control or NecJ-exposed CM did not affect α-SMA expression. These data suggest that ApoJ-exposed CM can suppress TGF-β1 induction of stress fibers and cytoskeletal changes that are essential for myofibroblast differentiation.

### Direct exposure of MLg cells to apoptotic cells does not inhibit myofibroblast phenotypic markers

In addition to macrophages and other professional phagocytes, fibroblasts can also clear apoptotic cells and this phagocytosis could modulate fibroblast proliferation and matrix deposition [19]. Thus, we examined whether CM from ApoJ-exposed MLg cells inhibited TGF-β1-induced fibroblast activation. Neither CM from ApoJ-exposed MLg cells nor direct exposure of MLg cells to apoptotic Jurkat cells inhibited TGF-β1-induced increases in α-SMA, type 1 collagen α2, or fibronectin expression (Supplementary Figure 2A-B). These data indicate that inhibition of TGF-β1-induced fibroblast activation requires bioactive mediators secreted by professional phagocytes after apoptotic cell stimulation.

### Interaction of macrophages and apoptotic cells antagonizes TGF-β1-induced activation of p38 MAP kinase, JNK, and Akt in MLg cells

**Figure 3:**
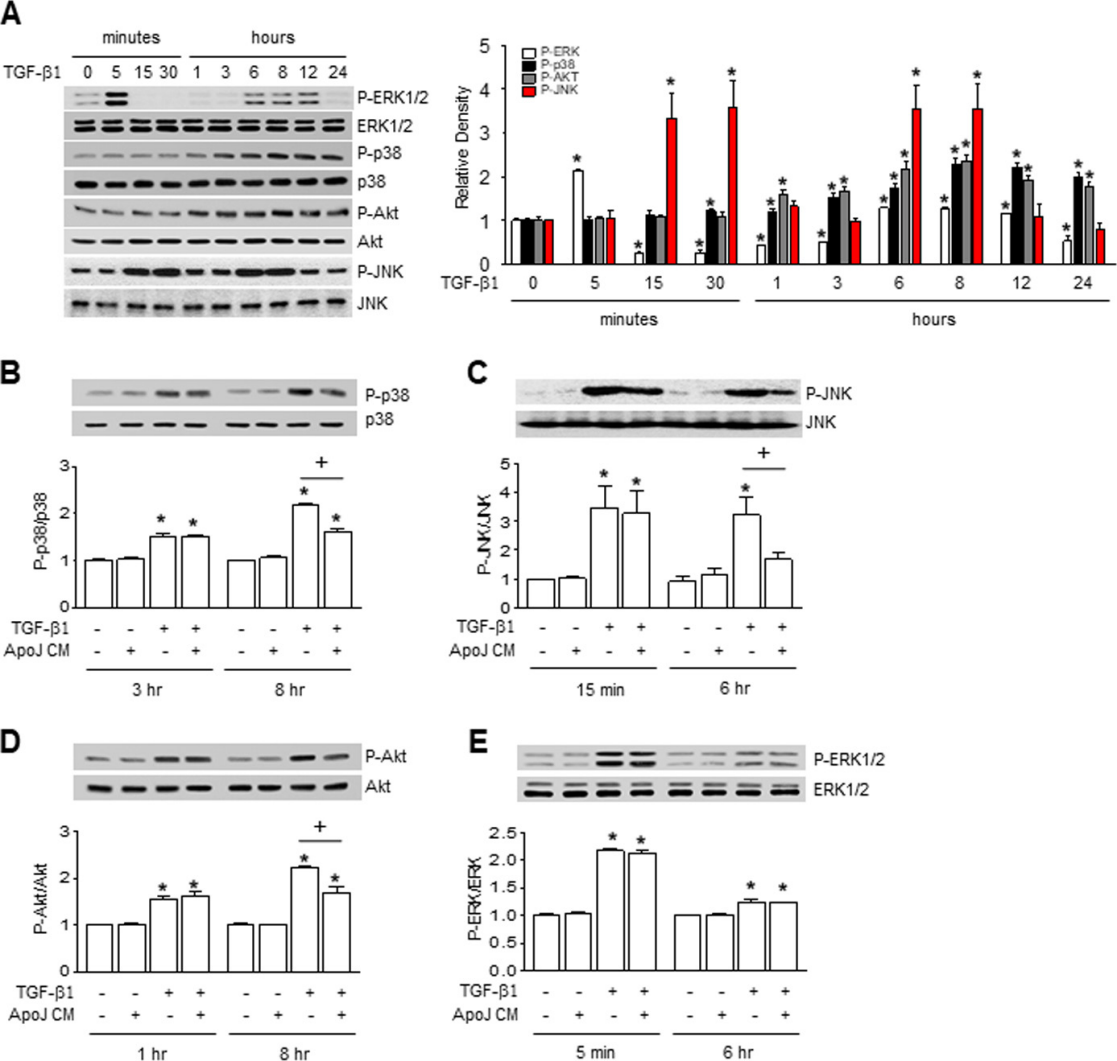
Conditioned medium from RAW 264.7 cells exposed to apoptotic cells blocked smad-independent TGF-β1 signaling in MLg cells RAW 264.7 cells were stimulated with apoptotic (ApoJ) for 20 h. Conditioned medium (CM) was added to MLg cells in the absence or presence of 10 ng/ml TGF-β1 for 24 h or the indicated time. (**A**–**E**) Western blot analysis of the relative amounts of total and phosphorylated ERK1/2, p38 MAP kinase, JNK1, and Akt protein in the indicated samples over time. Densitometric analysis of the relative phosphorylated protein abundances, normalized to that of total protein. Data in all bar graphs are the mean ± s.e.m. of three independent experiments. ^*^P < 0.05 compared with control; ^+^*P* < 0.05 as indicated.

Smad2 and Smad3 serve as the principle signaling transducers for TGF-β1 stimulation of profibrotic genes. However, the CM from ApoJ-exposed RAW 264.7 cells did not affect TGF-β1-mediated phosphorylation of Smad2 or Smad3 in MLg cells at 30 min and 1 h (Supplementary Figure 3A–3B). Recent studies have suggested that TGF-β1 responses can also be mediated by non-Smad signaling pathways, including mitogen-activated protein (MAP) kinase and phosphoinositide 3-kinase (PI3K)/protein kinase B (Akt) pathways [20]. We found that extracellular signal-regulated kinase (ERK) phosphorylation was substantially enhanced in MLg cells within 5 min of TGF-β1 stimulation. ERK levels then declined below the control at 3 h, but were slightly enhanced from 6–12 h. Furthermore, p38 MAP kinase and Akt phosphorylation were enhanced up to 24 h with the peak at 8 h (Figure 3A). c-Jun NH2-terminal kinase (JNK) 1 phosphorylation substantially enhanced at 15 and 30 min as well as 6 and 8 h after TGF-β1 stimulation. Interestingly, ApoJ-exposed CM treatment partially inhibited TGF-β1-induced phosphorylation of p38 MAP kinase, JNK1, and Akt; however, it had no effect on ERK phosphorylation (Figure 3B–3E). These data suggest that macrophages exposed to apoptotic cells secrete bioactive mediators that can partially block Smad-independent TGF-β1 signaling in MLg cells.

β1 responses can also be mediated by non-Smad signaling pathways, including mitogen-activated protein (MAP) kinase and phosphoinositide 3-kinase (PI3K)/protein kinase B (Akt) pathways [20]. We found that extracellular signal-regulated kinase (ERK) phosphorylation was substantially enhanced in MLg cells within 5 min of TGF-β1 stimulation. ERK levels then declined below the control at 3 h, but were slightly enhanced from 6–12 h. Furthermore, p38 MAP kinase and Akt phosphorylation were enhanced up to 24 h with the peak at 8 h (Figure 3A). c-Jun NH2-terminal kinase (JNK) 1 phosphorylation substantially enhanced at 15 and 30 min as well as 6 and 8 h after TGF-β1 stimulation. Interestingly, ApoJ-exposed CM treatment partially inhibited TGF-β1-induced phosphorylation of p38 MAP kinase, JNK1, and Akt; however, it had no effect on ERK phosphorylation (Figure 3B–3E). These data suggest that macrophages exposed to apoptotic cells secrete bioactive mediators that can partially block Smad-independent TGF-β1 signaling in MLg cells.

### COX-2-derived PGE_2_ and PGD_2_ in the CM inhibit MLg differentiation into myofibroblast

**Figure 4:**
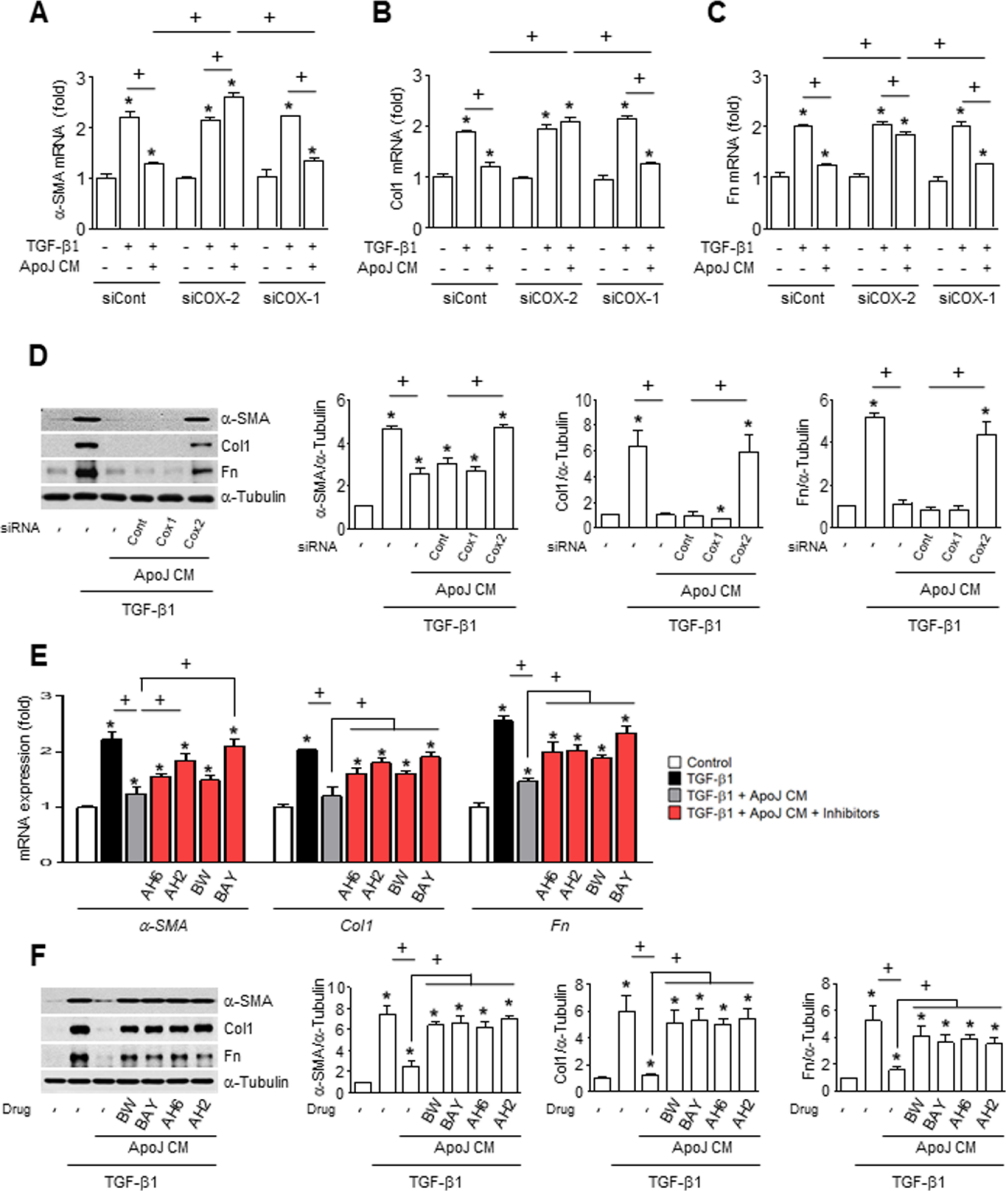
COX-2-derived PGE_2_ and PGD_2_ inhibit myofibroblast phenotype markers in MLg cells in conditioned medium (**A**–**D**) RAW cells were transfected with COX-2 siRNA or control vehicle for 6 h, then incubated with ApoJ for 20 h. Conditioned medium (CM) was added to MLg cells in the absence or presence of 10 ng/ml TGF-β1 for 24 h. (**E** and **F**) RAW 264.7 cells were stimulated with apoptotic Jurkat cells (ApoJ) for 20 h. CM was added to MLg cells in the presence of TGF-β1 with or without antagonists of EP2 (AH-6809), EP4 (AH-23848), DP1 (BW-A868C), or DP2 (BAY-u3405) at 10 mM. (A-C, E) The amount of myofibroblast phenotypic markers’ mRNA in MLg cell samples was analyzed by real-time PCR and normalized to that of *Hprt* mRNA. (D and F) Immunoblots of total cell lysates were performed with anti-α-SMA, type 1 collagen α2 (Col1), or fibronectin (Fn) antibodies. Right: Densitometric analysis of the indicated myofibroblast phenotypic markers’ relative abundances. Values represent the mean ± s.e.m. of three independent experiments. ^*^P < 0.05 compared with control; ^+^*P* < 0.05 as indicated.

Previously, we demonstrated that when exposed to apoptotic cells, RAW 264.7 cells produce cyclooxygenase-2 (COX-2)-derived PGE_2_ and PGD_2_ [21], which can inhibit myofibroblast differentiation and collagen secretion from lung fibroblasts [22, 23]. Thus, we examined whether COX-2 or COX-1 knockdown in macrophages would reverse the dampening effects of ApoJ-exposed CM on activated fibroblasts. We previously demonstrated that transfection of RAW 264.7 cells with COX-2 or COX-1 siRNA results in ~100 or 90% knockdown, respectively, at 20 h after ApoJ exposure compared to naïve RAW264.7 cells [15, 21]. Here, we demonstrate that similar knockdown of COX-2 reversed the ApoJ-exposed CM inhibition of TGF-β1-induced up-regulation of α-SMA, type 1 collagen α2, and fibronectin at both the mRNA (Figure 4A–4C) and protein level (Figure 4D). In contrast, COX-1 knockdown did not affect the TGF-β1-induced response of MLg cells with CM treatment.

To further examine the inhibitory effects of secreted PGE_2_ and PGD_2_ on TGF-β1-induced myofibroblast differentiation of MLg cells, ApoJ-exposed CM was added to TGF-β1-stimulated MLg cells in the presence of antagonists for E-prostanoid-2 receptor (EP2) (AH-6809), EP4 (AH-23848), DP1 (BW-A868C), or DP2 (BAY-u3405). The EP2 and DP1 antagonists only weakly affected TGFβ1-induced α-SMA mRNA expression, but significantly reversed the CM-induced reduction in type 1 collagen α2 and fibronectin mRNA expression (Figure 4E). In comparison, the EP4 and DP2 antagonists significantly reversed the ApoJ-exposed CM inhibition of TGFβ1-induced expression of all three myofibroblast phenotype markers at the transcript level. Interestingly, all these antagonists reversed the reduction of these markers at the protein level by ApoJ-exposed CM (Figure 4F).

### RhoA-dependent HGF secretion contributes to inhibition of myofibroblast differentiation by the CM

**Figure 5:**
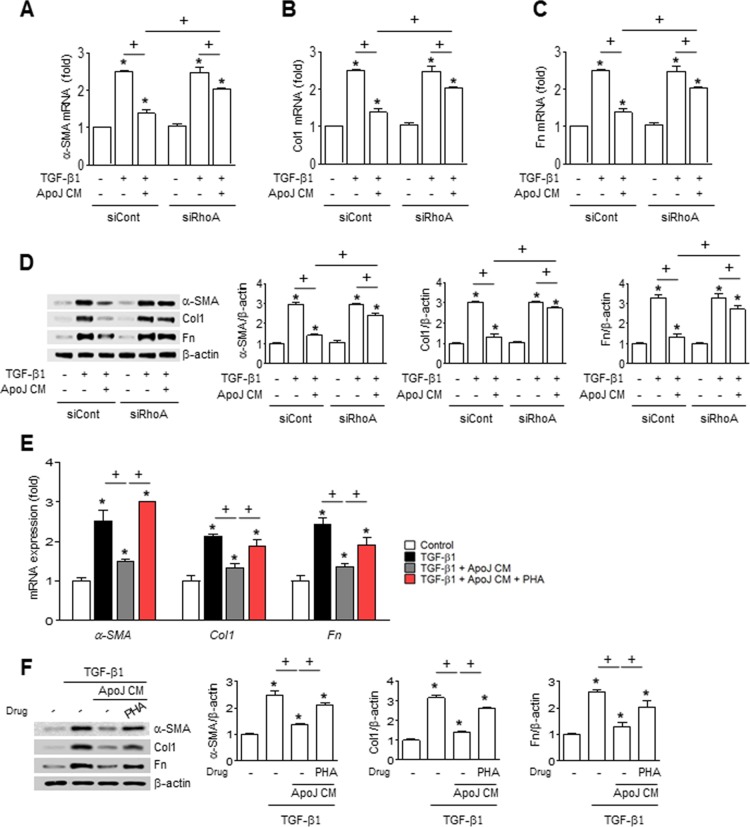
RhoA-dependent HGF secretion mediates the inhibition of myofibroblast phenotype markers in MLg cells by the conditioned medium (**A**–**D**) RAW 264.7 cells were transfected with RhoA siRNA or control vehicle for 24 h, then incubated with ApoJ for 20 h. Conditioned medium (CM) was added to MLg cells in the absence or presence of TGF-β1 for 24 h. (**E** and **F**) RAW 264.7 cells were stimulated with ApoJ for 20 h. CM was added to LA-4 cells in the presence of TGF-β1 with or without the antagonist of c-Met (250 nM PHA-665752). (A–C, E) The amount of myofibroblast phenotypic markers’ mRNA in MLg cell samples was analyzed by real-time PCR and normalized to that of *Hprt* mRNA. (D and F) Immunoblots of total cell lysates were performed with anti-α-SMA, type 1 collagen α2 (Col1), or fibronectin (Fn) antibodies. Right: Densitometric analysis of the indicated myofibroblast phenotypic markers’ relative abundances. Values represent the mean ± s.e.m. of three independent experiments. ^*^P < 0.05 compared with control; ^+^*P* < 0.05 as indicated.

Apoptotic cells induce HGF mRNA and protein production in macrophages via the RhoA-dependent pathway [24]. Thus, we examined whether RhoA-dependent HGF secretion from RAW 264.7 cells mediates the inhibitory effect of apoptotic cells on fibroblast activation. The ApoJ-exposed CM from RAW 264.7 cells transfected with RhoA siRNA reversed the reduction of TGF-β1-induced up-regulation of myofibroblast phenotype markers, including α-SMA, type 1 collagen α2, and fibronectin at the mRNA and protein level (Figure 5A–5D). Similarly, inhibition of HGF receptor (c-Met) signaling by PHA-665752 in MLg cells also reversed the reduction of these markers by ApoJ-exposed CM at the mRNA and protein level (Figure 5E–5F).

### Exogenous PGE_2_, PGD_2_, and HGF inhibit fibroblast differentiation into myofibroblasts

We next confirmed that PGE_2_, PGD_2_, and HGF act as paracrine factors to induce the anti-myofibroblast differentiation effect on MLg cells. The CM derived from RAW 264.7 cells without apoptotic cells contains basal levels of PGE_2_, PGD_2_ and HGF (50, 7, and 150 pg/ml, respectively), but reaches higher concentrations under stimulation conditions (150, 17, and 400 pg/ml, respectively) [15, 21]. As expected, basal concentrations of these soluble mediators alone or in combination did not inhibit TGF-β1-induced increases in protein expression for α-SMA, type 1 collagen α2, and fibronectin. However, stimulation concentrations of these bioactive molecules inhibited TGF-β1-induced changes in myofibroblast phenotypic markers (Supplementary Figure 4). Notably, we did not detect any synergistic effects when all three mediators were applied together at stimulation concentrations, suggesting that these factors may act on the same target to inhibit the TGF-β1-induced signaling pathway.

### Interaction of macrophages and apoptotic cells inhibits invasion of MLg cells

**Figure 6:**
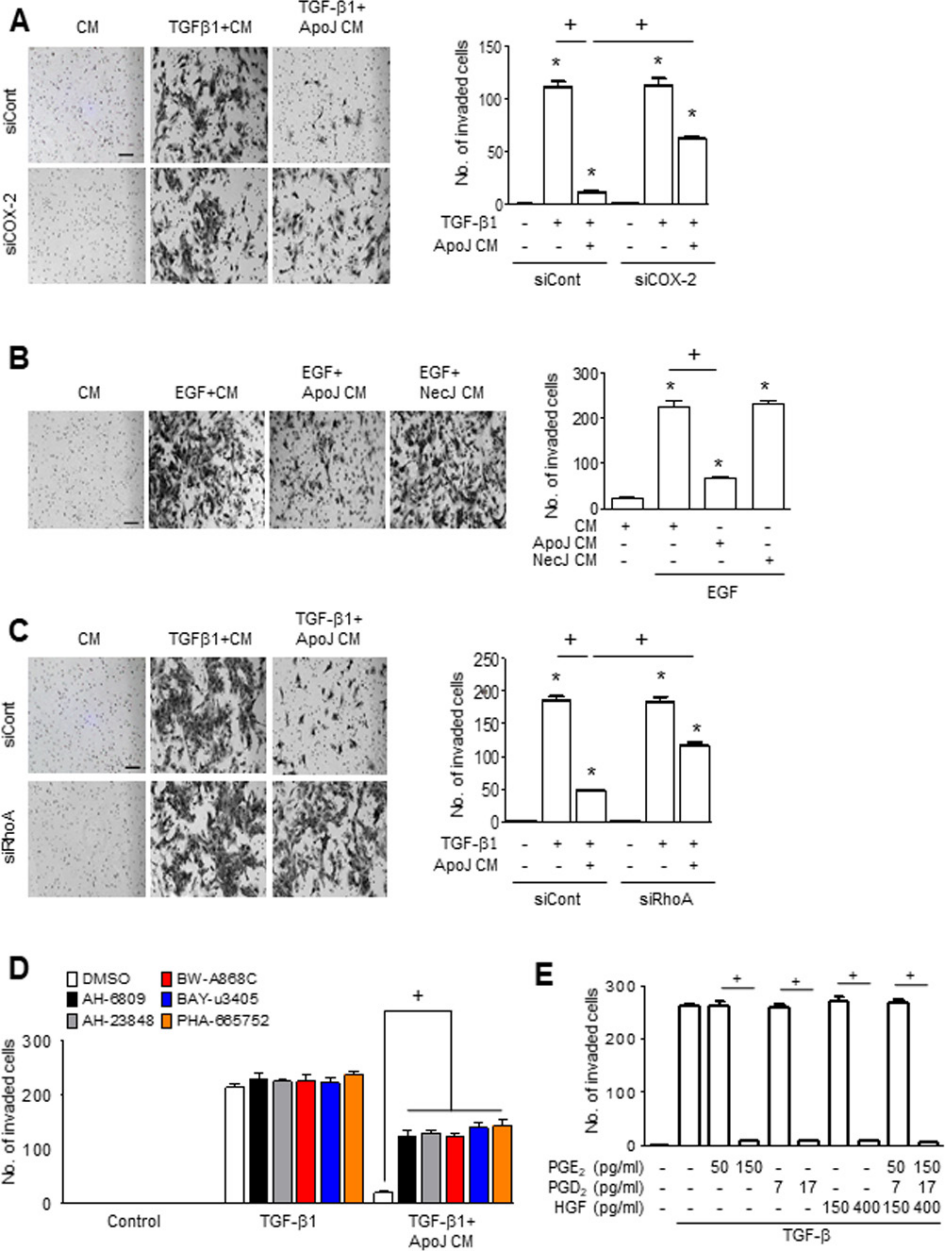
PGE_2_, PGD_2_, and HGF secretion mediates the inhibition of MLg cell invasion by the conditioned medium (**A** and **B**) Conditioned medium (CM) from RAW 264.7 cells exposed to apoptotic (ApoJ) or necrotic (NecJ) Jurkat cells for 20 h was added to MLg cells in the presence of 10 ng/ml TGF-β1 or EGF for 24 h. RAW cells were transfected with siRNA of COX-2 (A), RhoA siRNA (**C**), or control vehicle for 6 or 24 h, respectively, then incubated with ApoJ for 20 h. CM was added to MLg cells in the presence of 10 ng/ml TGF-β1 for 24 h. (**D**) RAW 264.7 cells were stimulated with ApoJ for 20 h. CM was added to MLg cells in the presence of TGF-β1 with or without antagonists of EP2 (AH-6809), EP4 (AH-23848), DP1 (BW-A868C), DP2 (BAY-u3405) at 10 mM, or PHA-665752 at 250 nM. (**E**) PGE_2_ (50 and 150 pg/ml), PGD_2_ (7 and 17 pg/ml), or HGF (150 and 400 pg/ml) was added to MLg cells in the presence of TGF-β1 for 48 h. (A–C) The cells were visualized by phase-contrast microscopy to analyze their invasive ability using Matrigel-coated Transwell. Scale bars: 100 μm. (A–E) Invaded cells were quantified by counting the number of cells adhering to the bottom surface of the upper chamber. Values represent the mean ± s.e.m. of three independent experiments. ^*^P < 0.05 compared with control; ^+^*P* < 0.05 as indicated.

Excessive invasiveness is also a hallmark of fibroblast activation and contributes to severe fibrotic lung disease [25, 26]. Therefore, we determined whether ApoJ-exposed CM could reduce the invasiveness of activated MLg cells. ApoJ-exposed CM treatment for 24 h inhibited the TGF-β1-induced invasion of MLg cells through a Transwell chamber coated with Matrigel matrix (Figure 6A). Similarly, epidermal growth factor (EGF) enhances MLg cell invasion through Matrigel, and this EGF-induced invasion was reduced by MLg treatment with CM from ApoJ-exposed, but not NecJ-exposed, RAW 264.7 cells (Figure 6B).

We next evaluated whether COX-2-derived PGE_2_ and PGD_2_ or RhoA-dependent HGF secretion from RAW 264.7 cells mediates the inhibitory effects of ApoJ-exposed CM on TGF-β1-induced MLg cell invasion. ApoJ-exposed CM from RAW 264.7 cells transfected with COX-2 or RhoA siRNA partially reversed the reduction of TGF-β1-induced increases in MLg cell invasion (Figure 6A and 6C, respectively). The anti-invasive effect of ApoJ-exposed CM was also prevented by treatment with antagonists of EP2, EP4, DP1, DP2, or c-Met (Figure 6D), suggesting that PGE_2_, PGD_2_, and HGF act on their respective receptors on MLg cells. Furthermore, exogenous PGE_2_, PGD_2_, and HGF at stimulation concentrations inhibited TGF-β1-induced invasive capacity of MLg cells, whereas basal concentrations of these bioactive molecules had no effect (Figure 6E). There was no synergistic effect on MLg cells when of all these macrophage-secreted mediators were added together at stimulation concentrations, suggesting that PGE_2_, PGD_2_, and HGF mediate the inhibition of TGF-β1-induced MLg cell invasion through shared downstream signaling pathways.

### *In vivo* exposure to apoptotic cells attenuates invasiveness of primary lung fibroblasts

**Figure 7:**
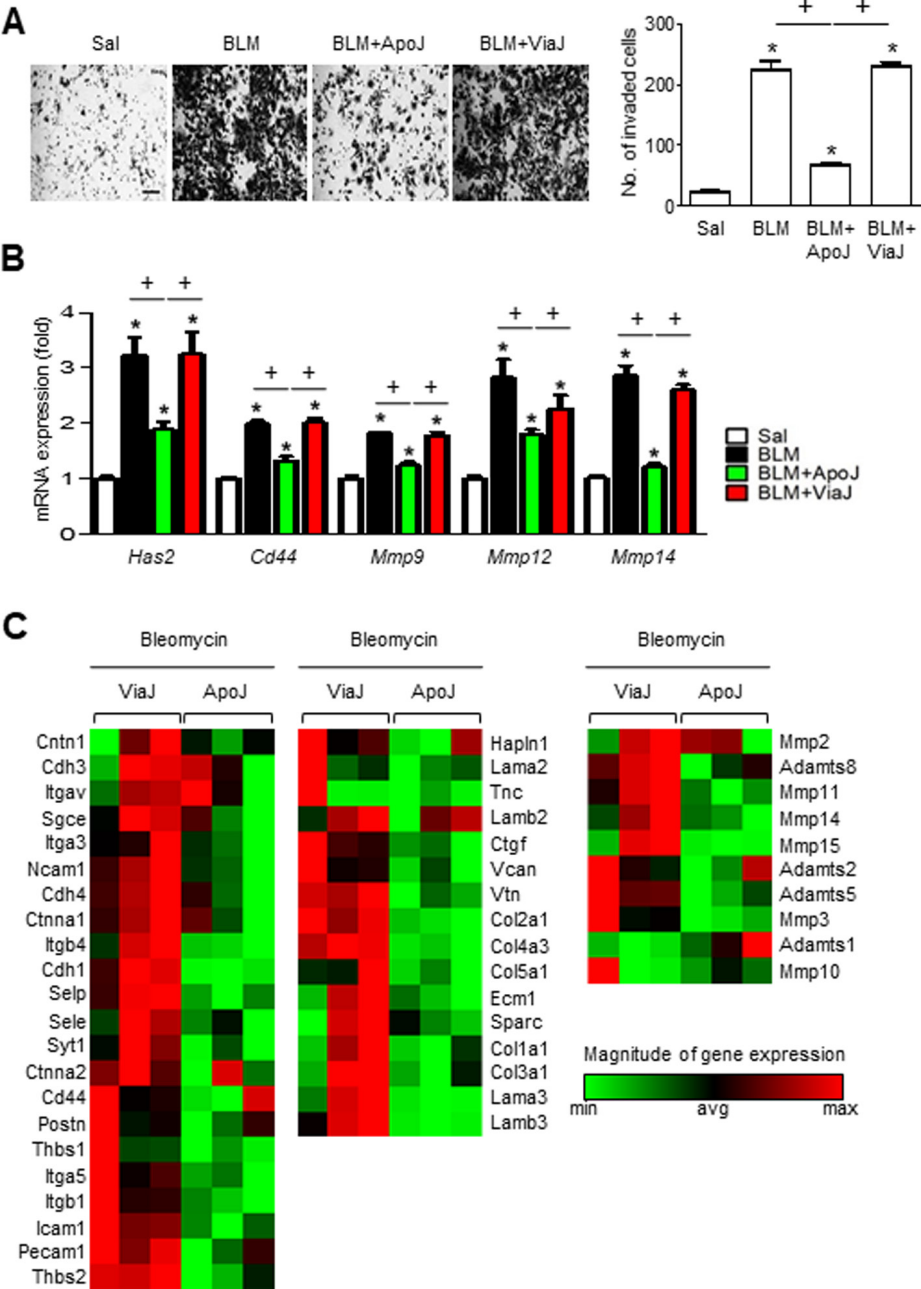
Apoptotic cell instillation suppresses invasiveness of primary lung fibroblasts and the expression of genes involved in invasion Two days after bleomycin (BLM) treatment, lungs were instilled with saline alone (Sal), viable Jurkat cells (ViaJ), or apoptotic Jurkat cells (ApoJ) intratracheally. Mice were euthanized at 7 days following BLM treatment. (**A** and **B**) Primary mouse fibroblasts were isolated from murine lungs. (A) The cells were visualized by phase-contrast microscopy to analyze their invasive ability using Matrigel-coated Transwell. Scale bar: 100 μm. *right side:* The invaded cells were quantified by counting the number of cells adhering to the bottom surface of the upper chamber. (B) mRNA expression profiles of hyaluronan synthase 2 (HAS2)*, CD44, MMP9, MMP12, and MMP14* in lung fibroblasts from each group was analyzed by real-time PCR. Values represent the mean ± s.e.m. of three independent experiments. ^*^P < 0.05 compared with control; ^+^*P* < 0.05 as indicated. (**C**) Selected heat maps showing differentially expressed genes for cell-cell/matrix adhesion (C, *left panel*) and ECM remodeling (C, *middle and right panels*) between BLM+ViaJ group and BLM+ApoJ group, with red indicating increased expression and green representing decreased expression versus the ViaJ control (*P* < 0.05, fold change > 2).

We assessed whether *in vivo* exposure to apoptotic cells could decrease the invasive capacity of isolated lung fibroblasts and protect from BLM-induced pulmonary fibrosis [12]. ApoJ cells were administered intratracheally two days after bleomycin (BLM) treatment and primary were fibroblasts were isolated at seven days after BLM treatment, which is when fibroblasts invasion peaks [25]. *In vivo* ApoJ treatment reduced the BLM-induced invasion of isolated fibroblasts through Matrigel (Figure 7A). However, there was no change in invasive capacity of fibroblasts from mice treated with BLM and viable Jurkat (ViaJ) cells. Progressive lung fibrosis requires fibroblast differentiation into an invasive myofibroblast phenotype, which is characterized by hyaluronan synthase 2 (HAS2) and CD44 expression and coordinated expression of matrix metalloproteinases (MMPs) and inhibitors of MMP functions [25]. In the present study, we investigated whether *in vivo* exposure to apoptotic cells suppresses the induction of HAS2, CD44, and MMPs in primary lung fibroblasts at seven days after BLM treatment. *In vivo* ApoJ administration inhibited BLM-induced increases in the mRNA levels of HAS and CD44, whereas viable cells had no effect (Figure 7B). Similarly, the BLM-induced mRNA up-regulation of MMPs that promote cell invasion, such as MMP9, MMP12, and MMP14, was reversed by *in vivo* administration of apoptotic cells. However, instillation of viable cells had little, if any, effect on mRNA abundances of these MMPs in primary lung fibroblasts. These data suggest that the presence of apoptotic cells in the lungs inhibits the invasive phenotype of activated lung fibroblasts through down-regulation of Has2 and CD44, as well as MMP9, MMP12, and MMP14.

### *In vivo* exposure to apoptotic cells alters expression of genes involved in cell adhesion and ECM remodeling

Gene expression signatures of invasive fibroblasts reveals upregulation of genes involved in cell adhesion and ECM remodeling, compared to non-invasive fibroblasts [25, 27]. To obtain additional insights into the mechanisms by which apoptotic cells can reduce fibroblast invasion, we analyzed 84 genes involved in cell adhesion and ECM remodeling using a targeted qPCR array. In the BLM+ApoJ group, 15 cell adhesion-related genes were strongly down-regulated compared to the BLM+ViaJ group. These genes included the following: *Ncam1*, *Cdh4*, *Itgb4*, *Cdh1*, *Selp*, *Sele*, *Syt1*, *Cd44*, *Postn*, *Thbs1*, *Itga5*, *Itgb1*, *Icam1*, *Pecam1*, and *Thbs2* (Figure 7C, *left panel*). In addition, ECM remodeling-related genes, such as ECM components (12 genes: *Ctgf*, *Vcan*, *Vtn*, *Col2a1*, *Col4a3*, *Col5a1*, *Ecm1*, *Sparc*, *Col1a1*, *Col3a1*, *Lama3*, and *Lamb3*) (Figure 7C, *middle panel*) and metalloproteinases (8 genes: *Adamts2*, *Adamts5*, *Mmp3*, *Mmp2*, *Adamts8*, *Mmp11*, *Mmp14*, and *Mmp15*) (Figure 7C, *right panel*), were also significantly down-regulated in the BLM+ApoJ group compared to those in the BLM+ViaJ group. These gene expression patterns were similar to those of non-invasive fibroblasts [27], suggesting that *in vivo* exposure to apoptotic cells inhibits the BLM-induced progression to an invasive myofibroblast phenotype.

## DISCUSSION

TGF-β signaling is a key mediator of fibroblast activation driving the aberrant synthesis of ECM in fibrotic diseases [17]. We previously demonstrated that apoptotic cell administration *in vivo* results in anti-inflammatory effect on BLM-treated lungs and prevents development of severe fibrosis [12, 14]. In the present study, we elucidated the cellular mechanism by which the interaction of macrophages with apoptotic cells inhibits lung fibroblast activation. Using *in vitro* models, we first demonstrated that the TGF-β1-induced increases in the expression of myofibroblast phenotypic markers, including α-SMA, type 1 collagen α2, and fibronectin, in MLg lung fibroblasts and primary isolated lung fibroblasts was inhibited by the addition of CM from macrophages exposed to apoptotic cells. Importantly, this inhibitory effect on MLg cell activation was observed for CM from primary murine BMDM exposed to apoptotic Jurkat cells and for RAW 264.7 cells exposed to multiple apoptotic cell types, including Jurkat cells, human HeLa epithelial cells, and primary mouse thymocytes. These data suggest that the macrophage response to apoptotic cells is universal and that the secreted mediators are cell-type independent. Moreover, the inhibition of mRNA and protein up-regulation of these myofibroblast markers did not occur when fibroblasts were incubated with CM from NecJ-exposed macrophages, indicating that the anti-fibrotic effects are specific for apoptotic cells. Similar to the anti-EMT effect of macrophages exposed to apoptotic cells [15], our data strongly suggest that trophic factors released by macrophages act in a paracrine manner on lung fibroblasts to inhibit the acquisition of a myofibroblast (fibroproliferative) phenotype.

β1-induced increases in the expression of myofibroblast phenotypic markers, including α-SMA, type 1 collagen α2, and fibronectin, in MLg lung fibroblasts and primary isolated lung fibroblasts was inhibited by the addition of CM from macrophages exposed to apoptotic cells. Importantly, this inhibitory effect on MLg cell activation was observed for CM from primary murine BMDM exposed to apoptotic Jurkat cells and for RAW 264.7 cells exposed to multiple apoptotic cell types, including Jurkat cells, human HeLa epithelial cells, and primary mouse thymocytes. These data suggest that the macrophage response to apoptotic cells is universal and that the secreted mediators are cell-type independent. Moreover, the inhibition of mRNA and protein up-regulation of these myofibroblast markers did not occur when fibroblasts were incubated with CM from NecJ-exposed macrophages, indicating that the anti-fibrotic effects are specific for apoptotic cells. Similar to the anti-EMT effect of macrophages exposed to apoptotic cells [15], our data strongly suggest that trophic factors released by macrophages act in a paracrine manner on lung fibroblasts to inhibit the acquisition of a myofibroblast (fibroproliferative) phenotype.

The effects of TGF-β are mediated by a complex network of intracellular signaling events. Smad proteins are considered to be the major signaling intermediaries for the stimulatory effects of TGF-β in fibroblasts [28]. However, the profibrotic effects of TGF-β activation can be mediated by smad-independent signal transducers, such as MAP kinases, PI3K/Akt [29], focal adhesion kinase, tyrosine kinase c-ABL, Wnt [30], and early growth response 1 [31]. In this study, we demonstrated that ApoJ-exposed macrophages secrete biological mediators that antagonize TGF-β1 signaling in MLg cells by partially blocking Smad-independent signaling pathways, such as p38 MAP kinase, JNK1, and PI3k/Akt, but have little effect on Smad-dependent signaling pathways and ERK1/2. However, the inhibition of the p38 MAP kinase, JNK1 and PI3K/Akt pathways was incomplete, suggesting that alternative signaling cascades are contributing to the inhibition of TGF-β1-mediated fibroblast activation by ApoJ-exposed CM.

We previously demonstrated that RAW 264.7 cells and primary peritoneal macrophages secrete COX-2-derived PGE_2_ and PGD_2_ and RhoA-dependent HGF in response to apoptotic cells [12, 21], and these bioactive mediators mediate an anti-EMT effect on fibroblasts [15]. In the present study, MLg treatment with exogenous PGE_2_, PGD_2_, or HGF reduced TGF-β1-induced increases in myofibroblast phenotypic markers. Furthermore, knockdown of COX-2 or RhoA expression in RAW 264.7 cells or MLg treatment with PGE_2,_ PGD_2_, or HGF receptor antagonists reversed the inhibitory effect of ApoJ-exposed CM on TGF-β1-induced MLg activation. Nonetheless, further studies will be needed to determine whether ectopic expression of COX-2 or RhoA in a COX-2^low^ or RhoA^low^ in a macrophage cell line can rescue the cells from EMT and fibrotic response. Similarly, others studies have demonstrated that PGE_2_ binding to EP2 on lung fibroblasts tempers the profibrotic, activating effects of TGF-β [32] and the migratory effects of fibroblast growth factor [33]. Additionally, PGD_2_ binding the DP receptor inhibits TGF-β-induced collagen secretion [23] and, in hepatic and renal fibrosis, HGF administration significantly reduced the deposition of extracellular matrix [34, 35]. Taken together, these data indicate that the PGE_2_-EP2/4, PGD_2_-DP1/2, and HGF-c-Met axes may prevent fibrosis by suppressing ECM production and inhibiting myofibroblast differentiation. Interestingly, addition of all these mediators together did not result in synergistic effects, suggesting that these signaling axes converge on the same target to inhibit TGF-β1-induced MLg activation.

Fibroblasts and myofibroblasts from IPF patients have been shown to have distinct properties [36], including the ability to invade extracellular matrix in a similar manner to metastatic cancer cells [37]. In addition to TGF-β1, other growth factors, such as EGF, fibroblast growth factor (FGF)-2, and platelet-derived growth factor (PDGF)-BB, have also been shown to induce fibroblast invasion [38]. PGE_2_ has been shown to increase PTEN activity and diminish bFGF-stimulated migration of human lung fibroblasts [33]. In addition, PGD_2_ signaling is involved in retinoid-inducible gene 1 (RIG1)-mediated suppression of testis cancer cell invasion [39]. In the present study, we demonstrated that ApoJ-exposed CM attenuated the enhanced invasive capacity of MLg cells stimulated with TGF-β1 or EGF. The anti-invasiveness effect was specific for macrophage recognition of apoptotic cells, because macrophage interaction with necrotic cells did not affect the invasive capacity of activated MLg cells. Similarly, the TGF-β1-induced invasive capacity of MLg cells was markedly abrogated by exogenous PGE_2_, PGD_2_, and HGF. Furthermore, the suppression of MLg invasion was reversed by MLg treatment with PGE_2_, PGD_2_, and HGF receptor antagonists or by decreased production of these factors via siRNA knockdown of COX-2 or RhoA in macrophages. These suggest that macrophage-secreted molecules act as paracrine factors to inhibit fibroblast invasion.

As an alternative approach, a synthetic peptide designed to disrupt the modulatory interaction of macrophages with apoptotic cells may inhibit transdifferentiation and invasion of lung fibroblasts. It is important to note that targeting tumor cells with CD47-specific blocking antibodies or soluble signal regulatory protein-α (SIRPα) variants inhibits CD47- SIRPα interaction and facilitates tumor cell removal by macrophages, leading to reduction tumor growth and metastasis [40–44]. Whether these synthetic peptides contribute to the anti-fibrogenic programming of macrophages through promoting engulfment of apoptotic cells warrants further investigation.

Previously, we also demonstrated that *in vivo* administration of apoptotic Jurkat T cells after BLM treatment reduced the mRNA expression of myofibroblast phenotypic markers in isolated primary lung fibroblasts [12]. Thus, in the present studies, we hypothesized that the interaction of macrophages with apoptotic cells *in vivo* would also reduce the invasive capacity of lung fibroblasts and impair the progression of lung fibrosis. Indeed, we demonstrated that primary fibroblasts from the BLM+ApoJ group were less invasive than fibroblasts from the BLM only treated group or the BLM+NecJ group. Taken together, our data provide *in vivo* evidence that apoptotic cell administration prevents fibroblast activation, transformation to myofibroblasts, and invasiveness in murine BLM-induced lung fibrosis.

Li and colleagues (2011) proposed that progressive lung fibrosis requires the generation of an activated myofibroblast phenotype that is characterized by overexpression of hyaluronan and HAS2 and the acquired ability to the invade basement membrane through the coordinated expression of the major cell surface receptor (CD44), MMPs, and inhibitors of MMP functions. In addition, CD44 has been shown to directly interact with MMP-14 (MT1-MMP) to promote tumor cell invasion [45]. Importantly, *in vivo* exposure to apoptotic cells into mouse lungs reduced the BLM-induced expression of HAS2, CD44, MMP9, MMP12, and MMP14 in primary isolated fibroblasts. Moreover, these isolated fibroblasts showed reduced expression of numerous genes related to cell adhesion to the ECM (15 genes) and ECM remodeling (20 genes). Interestingly, apoptotic cell instillation resulted in up-regulation of a disintegrin-like and metallopeptidase thrombospondin type 1 motif 1 (ADAMTS1), which has previously been shown to be down-regulated in invasive primary fibroblasts from bleomycin-treated lungs (Li *et al*. 2011). ADAMTS1 is a procollagen *N*-proteinase that processes several types of procollagen proteins and its down-regulation promotes progressive interstitial fibrosis in the kidney and fibroblast invasion in the lungs [25, 46]. Collectively, our data suggest that apoptotic cell recognition and clearance by macrophages is critical for blocking the generation of an invasive fibroblast phenotype and development of severe fibrosis.

Anti-inflammatory and anti-fibrotic action of apoptotic cells appears to conflict with a report by Wang *et al.* [47]. Where intratracheal instillation of apoptotic macrophages into untreated rat lungs resulted in inflammation, increased TNF-α and lung collagen, and enhances Sirius red staining. Differences between the two studies are: we used mice rather than rats; second, we used bleomycin-treated rather than untreated animals, and third, we used 10 × 10^6^ cells/mouse lung vs. 1 × 10^6^ cells/rat lung even though the mouse lung is 8 times smaller. The beneficial effects of cellular therapy using 5 × 10^6^ ~ 30 × 10^6^ apoptotic cells have already been evaluated in different murine models of acute and chronic inflammation, such as LPS-induced acute lung injury [48], sepsis [49], allergen-induced airway inflammation [50], inflammatory arthritis [51], and insulitis in mice type-1 diabetes [52], to restore or induce immune tolerance. In streptococcal cell wall (SCW)-induced arthritis in rats, 2 × 10^8^ apoptotic cells were used to suppress inflammation in joints and bones with local regulatory T-cell increase. Moreover, in this arthritis model, apoptotic cell injection alone (in the absence of SCW) did not induce any sign of arthritis occurrence. Further studies are required to understand the conflicting result and the effect on the anti-fibrotic hypothesis for apoptotic cells using different species treated with or without bleomycin.

In summary, our *in vitro* studies demonstrated that when macrophages interact with apoptotic cells, macrophages produce bioactive mediators that antagonize TGF-β1-induced myofibroblast (fibroproliferative) phenotypic markers and invasive capacity of lung fibroblasts. These macrophage-secreted bioactive mediators include the COX-2-derived PGE_2_ and PGD_2_ and the RhoA-dependent HGF. Our *in vivo* data demonstrated that apoptotic cell administration exerted an anti-invasiveness effect on primary fibroblasts by modulating the expression of genes related to cell-cell adhesion, cell-ECM adhesion, and ECM remodeling. Given the proximity of fibroblasts and macrophages in the fibrotic microenvironment, these findings present new insights into how changes in the lung microenvironmental influence the progression of an invasive myofibroblast phenotype. However, further studies will be required to confirm that bioactive mediators secreted by macrophages are diminishing lung fibroblast activation *in vivo*. More recently, the field has focused on addressing IPF not only as an epithelial-fibroblastic disorder, but also as a disease with many similarities to cancer progression [53]. Our data suggests that apoptotic cell therapies warrant further investigation as a tool for targeting fibroblasts and their cancer-like invasion mechanisms.

## MATERIALS AND METHODS

Detailed description for materials and methods, such as Reagents, Cell Culture, siRNA Transfection, Western Blotting, Real-Time Quantitative PCR (qPCR), Immunofluorescence, Invasion Assays, qPCR Array, and Statistics can be found in Supplementary materials.

### Isolation of primary BMDMs and lung fibroblasts

BMDM were differentiated from bone marrow myeloid stem cells of C57BL/6 mice as described previously [54]. After 7–10 days in culture with L929 complement DMEM, BMDM differentiation was confirmed by FACS analysis using anti-CD11b. Individual thymocytes were isolated from 3- to 4-week-old BALB/c mice by mincing the thymus through a cell strainer with a 70-μm pore size (BD Biosciences, Bedford, MA, USA). Primary murine lung fibroblasts were isolated from mice and purified using a modification of published methods [55]. In brief, lungs were perfused with 0.9% saline injected through the pulmonary artery until the lungs were cleared of blood. After lavage of lungs with 1 ml saline, dispase (100 units) was instilled into cleared mice lungs and the lungs were incubated for 45 min at room temperature (RT). Lung tissues were separated from large bronchi by mechanical means and transferred to a Petri dish containing DMEM with 0.01% DNase I for 10 min at 37°C. Lungs were cut into small pieces, minced, and digested enzymatically by DNase I in DMEM with 5% FBS for 90 min. After filtration (pore size 100 and 40 μm; SPL Life Sciences, Pocheon-si, Korea), cells were centrifuged, washed, and cultured for 3 days in 10 cm dishes in DMEM medium containing 10% FBS. Confluent cells at first passage were used for mRNA analysis.

### Induction of cell death

Human Jurkat T lymphocytes, human HeLa epithelial cells, and murine thymocytes were exposed to ultraviolet irradiation at 254 nm for 10 min followed by incubation in RPMI-1640 with 10% FBS for 2 h at 37°C and 5% CO_2._ Evaluation of nuclear morphology using light microscopy on Wright-Giemsa-stained samples indicated that the irradiated cells were approximately 70–80% apoptotic [21]. Lysed (necrotic) Jurkat T cells were obtained by multiple freeze-thaw cycles [56]. Apoptosis and necrosis were confirmed by Annexin V-FITC/propidium iodide (BD Biosciences, San Jose, CA) staining followed by flow cytometric analysis on a FACSCalibur system (BD Biosciences) [8].

### Co-incubation of macrophages with apoptotic or necrotic cells

Murine macrophages (RAW264.7 and BMDM) were plated at 5 × 10^5^ cells/ml and cultured overnight in suitable medium (detailed cell culture above) at 37°C and 5% CO_2_. The cells were then serum-starved with *X-VIVO* 10 medium (04-380Q, Lonza, Walkersville, MD, USA) for 24 h before stimulation. For the stimulation, the culture medium was replaced with *X-VIVO* 10 containing apoptotic or necrotic Jurkat cells, HeLa cells, or thymocytes (1.5 × 10^6^ cells/ml). After 20 h, supernatants were harvested, centrifuged, and filtered before being used as the conditioned medium for stimulation of target lung fibroblasts (5 × 10^5^ cells/ml).

### Incubation of fibroblasts with conditioned medium

MLg cells were plated in 6-well culture plates (2 × 10^5^ cells/well) and cultured overnight in 200 μl RPMI 1640 or DMEM, respectively, containing 10% FBS. Primary lung fibroblasts were plated and cultured on type 1 collagen-coated culture plates (1 × 10^6^ cells/well) for 48 h. Cells were treated for 24 h with conditioned medium from macrophages in the presence of 10 ng/ml TGF-β1. In some experiments, 10 mM AH-6809, AH-23848, BW-A868C, or BAY-u3405 or 250 nM PHA-665752 was used to antagonize EP2, EP4, DP1, DP2, or c-Met, respectively. The antagonist was added 1 h before the addition of the conditioned medium with 10 ng/ml TGF-β1.

### Mouse experiments

Specific pathogen free, male C57BL/6 mice (Orient Bio, Sungnam, Korea) weighing 20–22 g were used in all experiments. The Animal Care Committee of the EWHA Medical Research Institute approved the experimental protocol. Mouse pharyngeal aspiration was used for administration of the test solution [57]. Briefly, animals were anesthetized with a mixture of ketamine and xylazine (45 and 8 mg·kg^−1^ intraperitoneally, respectively), and placed individually on a board in a near-vertical position. The animal's tongue was extended with lined forceps. Sterile saline (0.9% sodium chloride) or 5 U·kg^−1^ body weight bleomycin in 30 ml saline was then placed posteriorly in the throat and aspirated into the lungs. Two days after BLM stimulation, saline alone or 1 × 10^7^ apoptotic or viable Jurkat T cells in 50 ml saline was administered intratracheally through pharyngeal aspiration [48]. Mice were sacrificed seven days after BLM treatment for isolation of primary lung fibroblasts.

### Statistics

Comparisons between two mean values ± SEM (control versus experimental) were performed using the two-tailed Student's *t*-test. *P* values less than 0.05 were considered statistically significant. All data were analyzed using GraphPad Prism 5 (La Jolla, CA, USA).

## SUPPLEMENTARY MATERIALS FIGURES



## Abbreviations

BLM: bleomycin
ApoJ: apoptotic Jurkat T lymphocyte cells
CM: conditioned medium
EGF: epidermal growth factor
ERK: extracellular signal-regulated protein kinase
HGF: hepatocyte growth factor
JNK: c-Jun NH2-terminal kinase
TGF-β: transforming growth factor-β
siRNAs: small interfering RNAs
