# Supplementary material for: Interaction of macrophages with apoptotic cells inhibits transdifferentiation and invasion of lung fibroblasts
Source: Oncotarget. 2017 Nov 28;8(68):112297–312. doi: 10.18632/oncotarget.22737 (PMC5762511; doi:10.18632/oncotarget.22737)
Supplement: Supplementary file 1 [file oncotarget-08-112297-s001.pdf]

Interaction of macrophages with apoptotic cells inhibits transdifferentiation and invasion of lung fibroblasts

SUPPLEMENTARY MATERIALS

Reagents

TGF-β1 (240-B-010) was from R&D Systems (Minneapolis, MN, USA). AH-23848, BW-A868C, BAY-u3405, PGE_2, PGD_2, and HGF were purchased from Cayman Chemical (Ann Arbor, MI, USA). AH-6809, bleomycin, and phalloidin (Sigma-Aldrich Chemical Co., St. Louis, MO), and PHA-665752 (Santa Cruz Biotechnology, Santa Cruz, CA, USA) were used as supplied. The gene-specific relative RT-PCR kit was obtained from Invitrogen (Carlsbad, CA, USA), and M-MLV reverse transcriptase was purchased from Enzynomics (Hanam, Korea). Primary antibodies used for Western blotting were as follows: ERK1/2 (sc-93-G), P-ERK1/2 (sc-7383), P38 (sc-535-G), P-P38 (sc-17852-R), AKT (sc-8312), P-AKT (sc-7985-R), JNK (sc-6254), P-JNK (sc-1648), Collagen (sc-393573) and α-Tubulin (sc-23948) antibodies were from Santa Cruz Biotechnology (Finnell Street Dallas, TX, USA). α-SMA, (ab7817), Fibronectin (ab2413) were from Abcam (Cambridge, MA, USA). Cleaved caspase-3 (D175, Cell Signaling) were from Cell Signaling Technology (Danvers, MA, USA). β-actin came from (Sigma-Aldrich Chemical Co., St. Louis, MO). Anti-mouse IgG-HRP (sc-2005), anti-rabbit IgG-HRP (sc-2004) and anti-goat IgG-HRP (sc-2354) used as secondary antibodies were purchased from Santa Cruz Biotechnology (Finnell Street Dallas, TX, USA).

Cell lines, primary cells, and culture

All cell lines were purchased from ATCC (American Type Culture Collection, Manassas, VA, USA) and primary macrophages or lung fibroblasts were obtained from C57BL/6 mice. Murine RAW 264.7 macrophages and MLg lung fibroblasts were grown in DMEM, (Gibco™, Thermo Fisher Scientific) supplemented with 10% FBS, and 1% penicillin/streptomycin. The human leukemia T cell line was maintained in RPMI 1640 (HyClone™, GE Healthcare) containing 10% fetal bovine serum (FBS) and 1% penicillin/streptomycin in a humidified incubator (37°C and 5% CO_2). For differentiation of monocytes to macrophages, bone marrow cells were cultured in DMEM supplemented with 10% FBS and 20% L929 supernatant (BMDM medium) for 7 days. Isolated lung fibroblasts were maintained in DMEM, (Gibco™, Thermo Fisher Scientific) supplemented with 10% FBS, and 1% penicillin/streptomycin for 3 days before the experiments in the Figure.

siRNA transfection

RAW 264.7 cells were transiently transfected with siRNA specifically targeting or control siRNA (SN-1003_AccuTarget™ Negative Control; Bioneer Inc) at both 100 nM final concentrations using GeneSilencer®siRNA Transfection Reagent (Genlantis Inc., San Diego, CA, USA) according to the manufacturer's instruction. Cells with the transfection mixture were incubated in serum-free medium for 6 h for COX-2 siRNA, 48 h for COX-1 siRNA, or 24 h for RhoA siRNA prior to experimentation. None of the siRNAs used had any significant effect on cell viability. After transfection, the cells were stimulated with apoptotic Jurkat T cells for 24 h. The siRNA sequences used for targeting genes were as follows (gene: sense, antisense). COX-2: 5′-CUAUGAUAGGAGCAUGUAA-3′, 5′-UUACAUGCUCCUAUCAUAG-3′; COX-1: 5′-GAGGUAGGAACUUUGACUA-3′, 5′-UAGUCAAAGUUCCUACCUC-3′; RhoA: 5′-GAA GUCAAGCAUUUCUGUCTTA-3′, 5′-GACAGAAAUGCUUGACUUCTT-3′.

Western blotting

Whole cell extracts were prepared from macrophages or target lung fibroblasts that had been exposed to apoptotic cells or conditioned medium (with or without pretreatment of pharmacological inhibitors for 1 hour). Cells were harvested, washed with ice-cold PBS, and lysed in Radioimmunoprecipitation assay buffer (RIPA buffer; 10 mM Tris, pH 7.2, 150 mM NaCl, 1% Nonidet P-40, 0.5% sodium deoxycholate, 0.1% SDS, 1.0% Triton

X-100, 5 mM EDTA) with protease inhibitors for 30 min on ice. Equal amounts of protein were resolved on SDS-PAGE gels (#161-0158, Bio-Rad Laboratories) and blotted on nitrocellulose membrane (10600001, GE Healthcare Life Science) using wet/tank transfer system (Bio-Rad Laboratories). After being blocked with 5% BSA-TBST or 5% Milk-TBST for 1 h, blots were incubated with the indicated primary antibodies for overnight.

Real-time quantitative PCR (qPCR)

Cells in the conditioned medium were grown to 80% confluence in triplicate on 6-well plates, washed twice with ice-cold PBS, and subjected to RNA extraction using 1 ml of Isol-RNA Lysis Reagent (5PRIME) according to the manufacturer's instruction. mRNA was reverse transcribed using the iScript^{TM} cDNA Synthesis Kit (BioRad). For quantitative PCR reactions, 1:5 dilution of cDNA products were amplified using SYBR Green PCR Master Mix (Applied Biosystems) and analyzed by using Real-Time PCR System (Applied Biosystems, Step One Plus). mRNA levels were normalized on the basis of HPRT mRNA. The primer sequences of target genes used were as follows (gene: forward, reverse): *α-SMA*: 5′-CCACCGCAAATGCTTCTAAGT-3′, 5′-GGCAGG AATGATTTGGAAAGG-3′; *Col1*: 5′-CAAGAAGAC ATCCCTGAAGTC-3′, 5′-ACAGTCCAGTTCTTCATT GC-3′; *Fn*: 5′-CACGATGCGGGTCACTTG-3′, 5′-CTG CAACGTCCTC CTCATTCTTC-3′; *Has2*: 5′-ACAGATG AGGCAGGGTCAAG-3′, 5′-TGGGGTGGAAAGAG AGAAGT-3′; *Cd44*: 5′-AGCGGCAGGTTACATTCAA A-3′, 5′-CAAGTTTTGGTGGCACACA G-3′; *Mmp9*: 5′-T GCCCAGCGACCACAACTC-3′, 5′-CGGACCCGAAGC GGACATT-3′; *Mmp12*: 5′-TTTGGATTATTGGAATGCT GC-3′, 5′-ATGAGGCAGAAACGTGGACT-3′; *Mmp14*: 5′-GTGAGCGTTGTGTGTGGGTA-3′, 5′-CCCAAGGCA GCAACTTCAG-3′.

Immunofluorescence

Differentiation to myofibroblasts in MLg cells grown on glass coverslips was visualized via immunocytochemistry with fibrosis markers. After the incubation period under experimental conditions, cells were washed twice in cold PBS before fixation with 4% paraformaldehyde (PFA) solution for 8 min at room temperature (RT). After fixation, cells were washed three times with PBS for 5 min each and incubated with 0.5% Triton X-100 in PBS at RT for 5 min. After subsequent rinsing with PBS, cells were treated with the blocking buffer containing 5% BSA and 5% normal goat serum for 1 h at RT, and incubated with α-SMA antibody for overnight at 4°C. After PBS washing in the same way as described above, cells were treated with secondary antibodies for 1h at RT and washed three times with PBS for 5 min each. For a double staining on filamentous actin and a-SMA, Phalloidin (P5282, Sigma-Aldrich) was diluted in PBS (dilution ratio; 1:50) and put on the slides for 30 min at RT. Anti-fade mounting was performed using VECTASHIELD Mounting Medium with DAPI (H-1200, Vector Laboratories) after a rinse with distilled water followed by the image collections using a Carl Zeiss LSM 800 confocal microscope.

Invasion assays

Cell invasion was tested using Transwell chambers (Corning Inc) coated with Matrigel matrix of 700 μg/ml for MLg cells or 200 μg/ml for primary fibroblasts according to the manufacturer`s instruction. In brief, pre-incubated lung fibroblasts (2×10^4 MLg cells/well or 1×10^5 primary cells/well) in the conditioned medium from macrophages in the absence or presence of 10 ng/ml TGF-β1 were plated in replicate wells in serum-free DMEM in the upper chambers and in DMEM supplemented with 10% FBS placed in the bottom wells at 37°C for 24 h. After fixation in 4% paraformaldehyde, the noninvaded cells on the upper surface of the membrane were scraped off with a cotton swap. The cells on the lower surface were stained using 0.5% crystal violet, and washed with distilled water. Three random microscopic fields from replicate wells per sample (10X magnification) were photographed and counted.

qPCR array

RNA of fibroblasts was isolated from bleomycin-treated male C57BL/6 mice in the same way as described in Real-Time Quantitative PCR (qPCR). Reverse transcription was performed with the RT2 First Strand cDNA Synthesis kit (330401, QIAGEN), and 84 genes were assessed by qPCR using the Mouse Extracellular Matrix and Adhesion Molecules array (RT2 ProflerTM PCR Array; PAMM-013ZC-12, QIAGEN) according to the manufacturer's instructions using the ABI StepOnePlusTM. For analysis, the expression level for each gene of interest (GOI) was calculated as 2-Ct followed by normalization to GAPDH, the housekeeping gene (HKG), with the formula 2-(Ct GOI - Ct HKG). Ultimately, the fold change in normalized gene expression was calculated by comparing values from fibroblasts purified from EXP mice (bleomycin+ApoJ group) to those purified from CTL mice (bleomycin+ViaJ group) according to the following formula: $2^{-\Delta Ct\ EXP}/2^{-\Delta Ct\ CTL.}$ Values were calculated for three mice per group, and genes were regarded as significantly changed if the P value was <0.05 and fold was >2.

Statistics

Comparisons between 2 mean values ± SEM (control versus experimental) were performed using the two-tailed Student's *t*-test. *P* values that are less than 0.05 are considered statistically significant. All data were analyzed using Graph Prism 5 software. (GraphPad Software Inc., La Jolla, CA, USA).

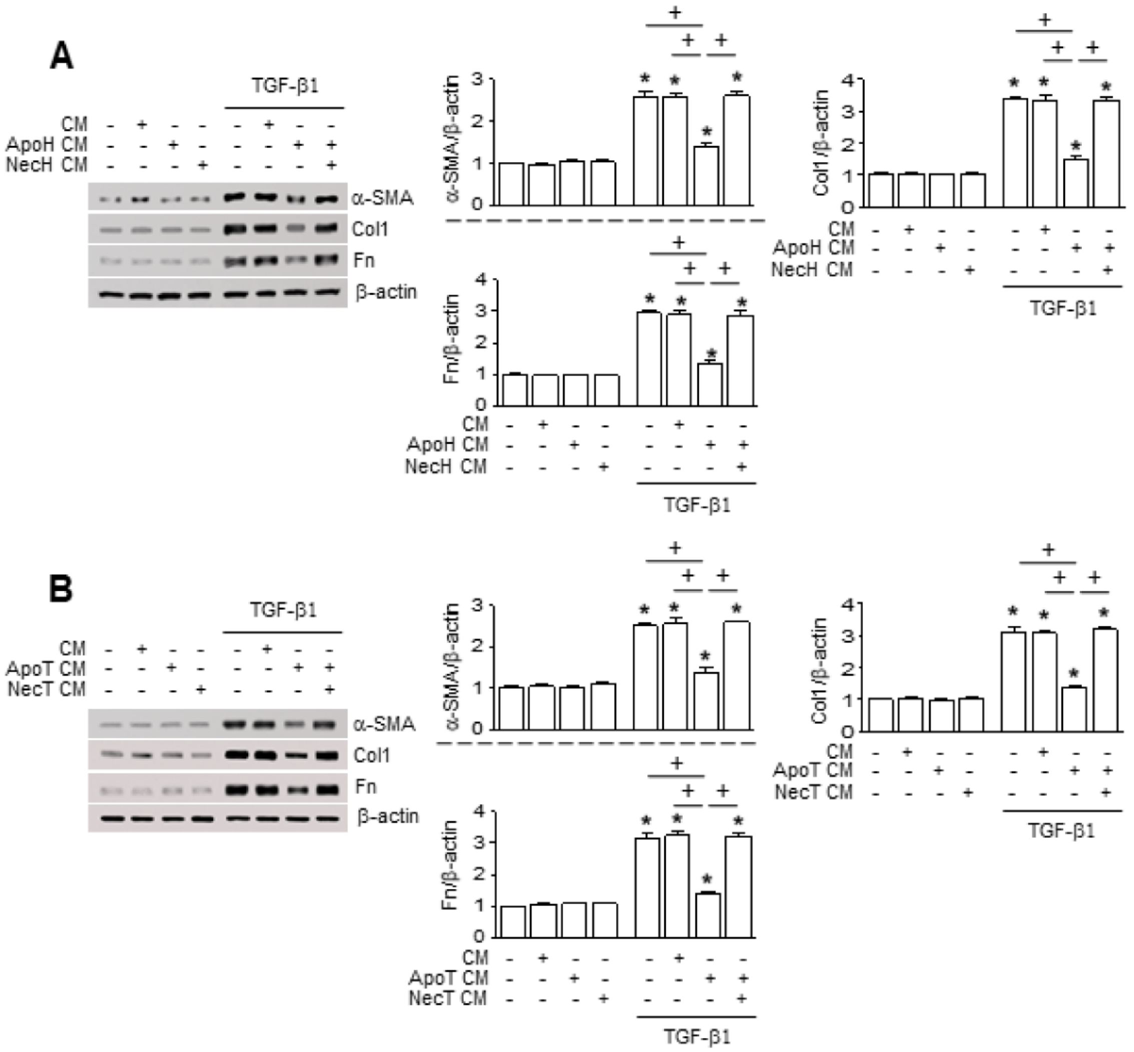

Supplementary Figure 1: Conditioned medium from macrophages exposed to apoptotic cells reduces TGF-β1-induced myofibroblast phenotypic marker expression in lung fibroblasts. RAW 264.7 cells were stimulated with apoptotic or necrotic HeLA cells (ApoH, NecH) or primary thymocytes (ApoT, NecT) for 20 h. Conditioned medium (CM) was added to MLg cells in the absence or presence of 10 ng/ml TGF-β1 for 24 h. (**A**, **B**) Immunoblots of total cell lysates were performed with anti-α-SMA, type 1 collagen α2 (Col1), or fibronectin (Fn) antibodies. Right: Densitometric analysis of the indicated myofibroblast phenotypic markers' relative abundances. Values represent the mean ± s.e.m. of three independent experiments. $^{*}P < 0.05$; compared with control; $^{+}P < 0.05$ as indicated.

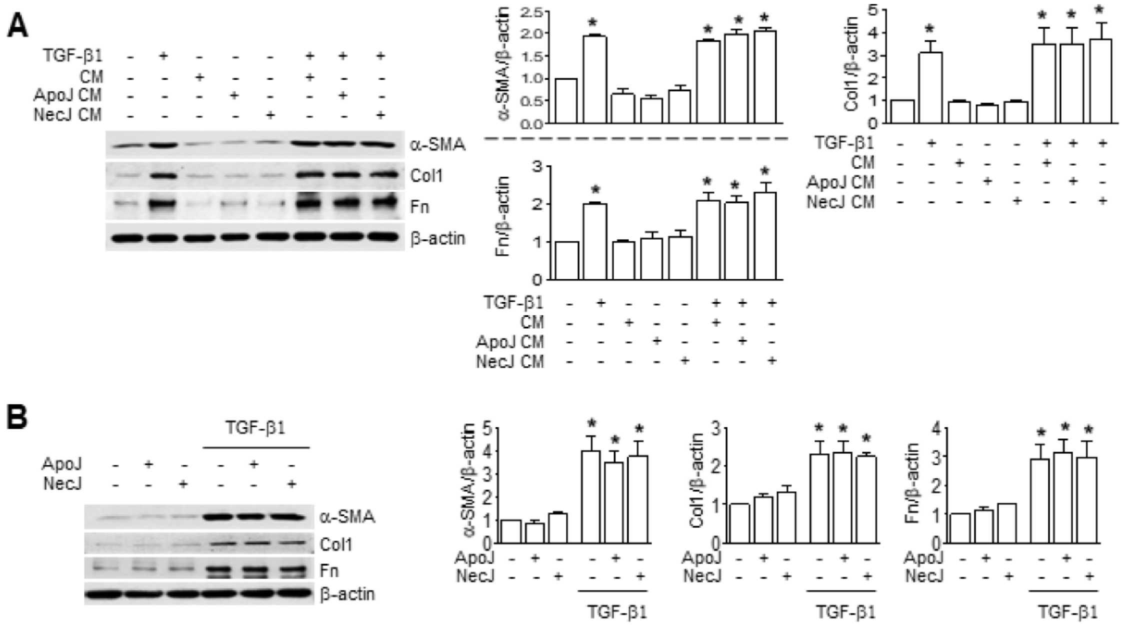

Supplementary Figure 2: Effect of direct exposure of MLg cells to apoptotic cells on TGF-β1-induced myofibroblast phenotypic marker expression. (**A**) MLg cells were stimulated with apoptotic (ApoJ) or necrotic (NecJ) Jurkat cells for 20 h. Conditioned medium (CM) was added to MLg cells in the absence or presence of 10 ng/ml TGF-β1. (**B**) MLg-4 cells were directly exposed to ApoJ or NecJ cells in the absence or presence of 10 ng/ml TGF-β1. (A, B) After 24 h, Immunoblots of total cell lysates were performed with anti-α-SMA, type 1 collagen α2 (Col1), or fibronectin (Fn) antibodies. Right: Densitometric analysis of the indicated myofibroblast phenotypic markers' relative abundances. Values represent the mean ± s.e.m. of three independent experiments. $^{*}P < 0.05$; compared with control; $^{+}P < 0.05$ as indicated.

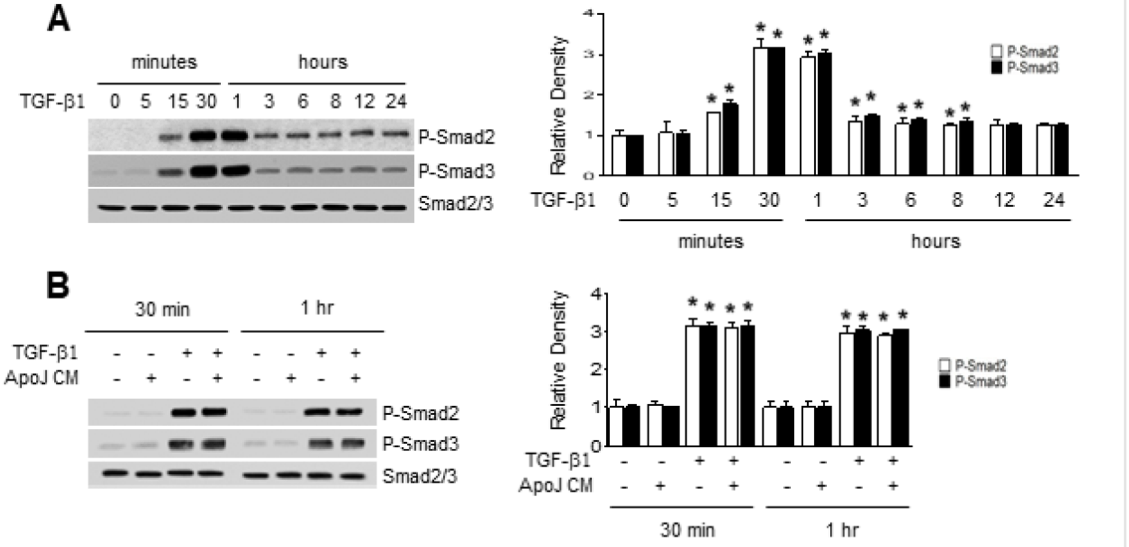

Supplementary Figure 3: Conditioned medium from RAW 264.7 cells exposed to apoptotic cells did not block Smad-dependent TGF-β1 signaling in MLg cells. RAW 264.7 cells were stimulated with apoptotic Jurkat cells (ApoJ) for 20 h. Conditioned medium (CM) was added to Mlg cells in the absence or presence of 10 ng/ml TGF-β1 for the indicated time. (**A**, **B**) Western blot analysis of the relative amounts of total and phosphorylated Smad2, Smad3 protein in the indicated samples over time. Densitometric analysis of the relative phosphorylated protein abundances, normalized to that of total protein. Data in all bar graphs are the mean ± s.e.m. of three independent experiments. $^{*}P < 0.05$ compared with control.

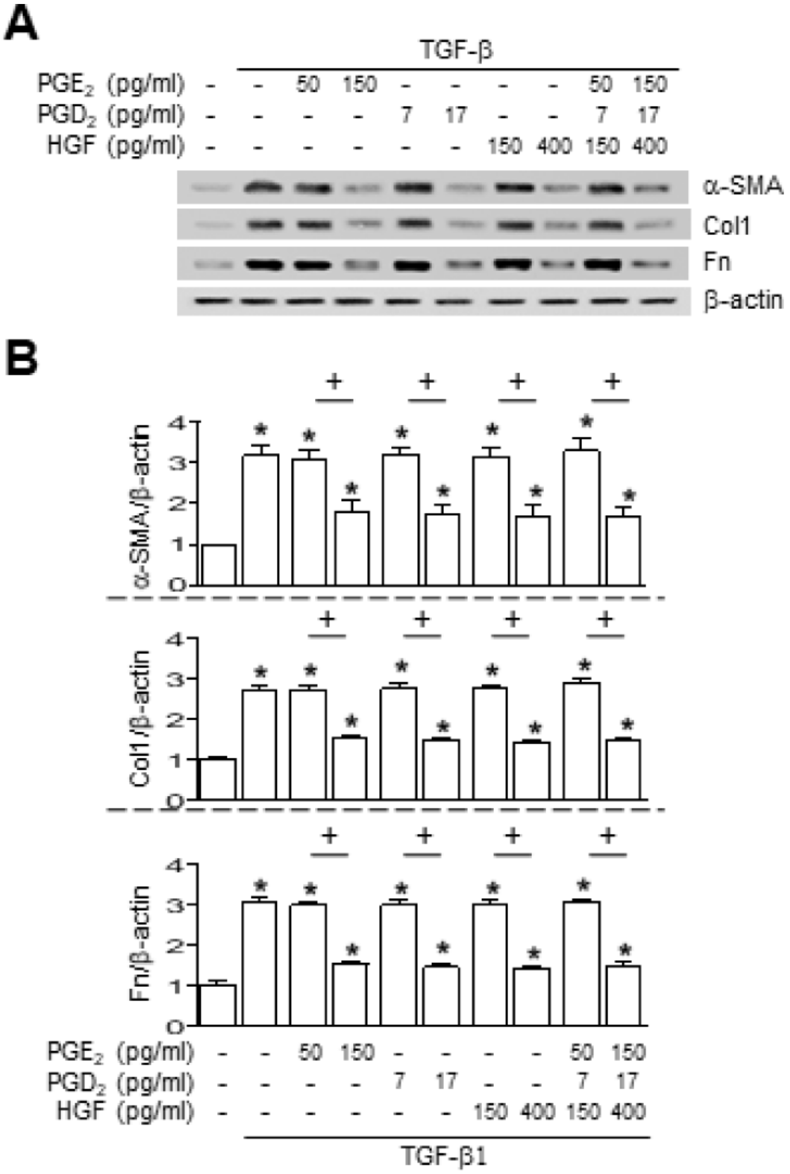

Supplementary Figure 4: Exogenous PGE_2, PGD_2, and HGF mediate reduction of myofibroblast phenotypic markers in MLg cells. PGE_2 (50 and 150 pg/ml), PGD_2 (7 and 17 pg/ml), and HGF (150 and 400 pg/ml) were added individually or all together to MLg cell culture in the presence of TGF-β1 for 24 h. (**A**) Immunoblots of total cell lysates were performed with anti-α-SMA, type 1 collagen α2 (Col1), or fibronectin (Fn) antibodies. (**B**) Densitometric analysis of the indicated myofibroblast phenotypic markers' relative abundances. Values represent the mean ± s.e.m. of three independent experiments. $^{*}P < 0.05$; compared with control; $^{+}P < 0.05$ as indicated.
